# Supplementary material for: mastermind regulates niche ageing independently of the Notch pathway in the Drosophila ovary
Source: Open Biol. 2019 Nov 20;9(11):190127. doi: 10.1098/rsob.190127 (PMC6893403; doi:10.1098/rsob.190127)
Supplement: Supplementary Material [file rsob190127supp1.pdf]

SUPPLEMENTARY MATERIAL FOR:

mastermind regulates niche ageing independently of the *Notch* pathway in the *Drosophila* ovary.

María Lobo-Pecellín, Miriam Marín-Menguiano and Acaimo González-Reyes*
*Correspondence to: AG-R (agonrey@upo.es)

Supplementary Material and Methods

Supplementary References

Supplementary table:

Table S1 (related to Figures 1 and S1).

Supplementary figure legends

Figure S1 (related to Figure 1).

Figure S2 (related to Figure 2).

Figure S3 (related to Figures 2 and 3).

Figure S4 (related to Figure 3).

Figure S5 (related to Figure 4).

Figure S6 (related to Figure 5).

Figure S7 (related to Figure 6).

Supplementary Materials and Methods

Genotypes

Figure 1

(B, C) *y w*

(D) *bab1>tau::GFP*: *w;; bab1-Gal4*, *UAS-tau::GFP/TM2*

Figure 2

(A) *w;; bab1-Gal4*, *UAS-tau::GFP/TM2*

(B) *mam* enhancer>*tau::GFP: w;; mam(GMR28A08)-Gal4/UAS-tau::GFP*

(C, D) Control: *w; UAS-mam/+*; *TM6B/+*

bab1>mam: *w; UAS-mam/+*; *bab1-Gal4*, *tub-G80*^{ts}*/+*

(E) *hh>tau::GFP*: *w*; *tub-G80*^{ts}*/+; hh-Gal4/UAS-tau::GFP*

(F) *hh>mam*: *w*; *UAS-mam/tub-G80*^{ts}; *hh-Gal4/+*

(G) *ptc>tau::GFP*: *w*; *tub-G80*^{ts}*/+; ptc-Gal4/UAS-tau::GFP*

(H) *ptc>mam*: *w*; *UAS-mam/tub-G80*^{ts}; *ptc-Gal4/+*

(F, H) Control: *w; UAS-mam/tub-G80*^{ts}; *TM6B/+*

Figure 3

(A) *y w*; D*E-cadherin::GFP;* MKRS/TM6B

(C) Control: *w*; *UAS-mam*/D*E-cadherin::GFP*; *TM6B/+*

bab1>mam: w; *UAS-mam/* D*E-cadherin::GFP*; *bab1-Gal4, tub-G80*^{ts}*/+*

Figure 4

(A) *y w*; D*E-cadherin::GFP;* MKRS/TM6B

(B) *w*; *Resille::GFP*

(E) *y w*

(G) Control: *w; UAS-mam/+*; *TM6B/+*

bab1>mam: w; UAS-mam/+; *bab1-Gal4*, *tub-G80*^{ts}*/+*

Figure 5

(A, E, F) *y w*

(C, G) Control: *w; UAS-mam/+; TM6B/+*

bab1>mam: w; UAS-mam/+; bab1-Gal4, tub-G80^{ts}*/+*

Figure 6

(A) Control: *w; UAS-mam/UAS-SOD1; TM2/TM6B*

bab1>mam+GFP: w; UAS-mam/+; UAS-tau::GFP/bab1-Gal4, tub-G80^{ts}

bab1>mam+SOD1: w; UAS-mam/UAS-SOD1; bab1-Gal4, tub-G80^{ts}*/TM2*

(B-D) Control: *w; UAS-Dicer/+; UAS-(Dl RNAi or N*^{intra}*)/TM2*

bab1>Dl RNAi: w; UAS-Dicer/+; UAS-Dl RNAi/bab1-Gal4, tub-G80^{ts}

bab1>N^{intra}*: w; UAS-N*^{intra}*/+; bab1-Gal4, tub-G80*^{ts}*/TM2*

bab1>mam: w; UAS-mam/+; bab1-Gal4, tub-G80^{ts}*/+*

Suppl. Figure 1

w;; bab1-Gal4, UAS-tau::GFP/TM2

Suppl. Figure 2

(A, B) Control: *w; UAS-dicer/+; bab1-Gal4, tub-G80*^{ts}*/TM2*

bab1>mam RNAi: w; UAS-dicer/+; bab1-Gal4, tub-G80^{ts}*/UAS-mam RNAi*

(C) *y w/w; TM6B/+*

y w/w; bab1-Gal4, tub-G80^{ts}*/+*

(D) Control: *w; tj-Gal4/+; tub-G80*^{ts}/TM2

bab1>mam^{Short}*: w;; bab1-Gal4, tub-G80*^{ts}*/UAS-mam*^{Short}

(E) Control: *w; UAS-mam*^{Short}*/TM6B*

tj>mam^{Long}: *w; UAS-mam*^{Long}*/tj-Gal4; tub-G80*^{ts}/TM2

(F) *w; tub-G80*^{ts}*/+; TM6B/+*

w; tub-G80^{ts}*/+; hh-Gal4/+*

w; tub-G80^{ts}*/+; ptc-Gal4/+*

Suppl. Figure 3

(A) *y w*

(B) Control: *w; UAS-mam/+*; *TM6B/+*

bab1>mam: w; UAS-mam/+; *bab1-Gal4*, *tub-G80*^{ts}*/+*

Suppl. Figure 4

(A, B) Control: *w; UAS-mam/+*; *TM6B/+*

bab1>mam: w; UAS-mam/+; *bab1-Gal4*, *tub-G80*^{ts}*/+*

(C, D) *w*; *UAS-mam/+*; *UAS*-D*E-cadherin/TM6B*

w; *UAS-nls::GFP/+*; *UAS*-D*E-cadherin/TM6B*

*bab1> GFP+*D*E-cad: w*; *UAS-nls::GFP/+*; *UAS*-D*E-cadherin/bab1-Gal4, tub-G80*^{ts}

bab1>mam+GFP: w; *UAS-mam/UAS-nls::GFP*; *bab1-Gal4, tub-G80*^{ts}*/TM2*

*bab1>mam+*D*E-cad: w*; *UAS-mam/+*; *UAS*-D*E-cadherin/bab1-Gal4*, *tub-G80*^{ts}

Suppl. Figure 5

(A) Control: *w*; *UAS-mam/*D*E-cadherin::GFP*; *TM6B/+*

bab1>mam: w; *UAS-mam/* D*E-cadherin::GFP*; *bab1-Gal4*, *tub-G80*^{ts}*/+*

(C) *y w*

(E) Control: *w; UAS-mam/+*; *TM6B/+*

bab1>mam: w; UAS-mam/+; *bab1-Gal4*, *tub-G80*^{ts}*/+*

Suppl. Figure 6

(A) *y w*

(C) Control: *w; UAS-mam/+*; *TM6B/+*

bab1>mam: w; UAS-mam/+; *bab1-Gal4*, *tub-G80*^{ts}*/+*

(E) *bab1>hh::GFP: w; UAS-hh::GFP/Sp*; *bab1-Gal4, tub-Gal80ts/*+

Control: *w; UAS-hh::GFP/UAS-mam*; +/TM6B

bab1>mam+GFP: w; UAS-nls::GFP/UAS-mam; *bab1-Gal4, tub-Gal80ts/*+

bab1>mam+hh: w; UAS-hh::GFP/UAS-mam; *bab1-Gal4, tub-Gal80ts/*+

Suppl. Figure 7

(A, B) Control: *w*; *UAS-mam/UAS-SOD1; TM2/TM6B*

bab1>mam+SOD1: w; *UAS-mam/UAS-SOD1; bab1-Gal4*, *tub-G80^{ts}/TM2*

(C) Control: *w; UAS-SOD1*/CyO; *TM6B/TM2*

bab1>SOD1: w; UAS-SOD1/CyO; *bab1-Gal4*, *tub-G80^{ts}/TM6B*

Fly stocks

Flies were grown on standard medium at 18, 25 or 29°C, depending on the experiment. To overexpress GFP in niche cells, we used *bab1-Gal4* [1], *mam-Gal4* (BDSC 49444; it expresses Gal4 under the control of DNA sequences in or near *mam; GMR28A08), hh-Gal4* [2] or *ptc-Gal4* (BDSC 45900) in combination with *UAS-tau::GFP* [3] or *UAS-nls::GFP* (BDSC 4775). The *D*E-cadherin::GFP line (BDSC 60584) is a genetically modified version of the endogenous *shotgun* gene, engineered to express a GFP-tagged E-cadherin under its native regulatory sequences [4]. As a membrane marker for live imaging we used Resille::GFP [5]. For overexpression experiments we used the following lines: *UAS-hh::GFP [6], UAS-mam* (long isoform; BDSC 27743)*, UAS-mam^{Short}* [7]*, UAS-N^{intra}* (BDSC 52008), *UAS-SOD1* [8] (BDSC 33605) or *UAS*-D*E-cadherin* [9] in combination with *tub-Gal80^{ts}* (BDSC 7017) and *bab1-Gal4* [1] or *traffic-jam-Gal4* [10]. To knock-down *mam* or *Delta* levels we utilised *UAS-mam RNAi* (BDSC 28046) and *UAS-Delta RNAi* (BDSC 34938) in combination with *UAS-Dicer* (BDSC 24650), *bab1-Gal4* and *tub-Gal80^{ts}*. Where necessary, flies of the appropriate genotype were shifted from 18°C to 29°C for 1 or 2 weeks upon eclosion and prior to dissection.

Immunohistochemistry

Most antibody stainings were performed as previously reported [11] with minor modifications. Ovaries were fixed with 1xPBS + 0.1% Tween-20 + 4% Formaldehyde (FA), washed with 1xPBS + 0.1% Triton X-100 (PBS-T), permeabilized for 1 hour with 1xPBS + 1% Triton X-100 and blocked for 1 hour with 1xPBS + 0.1% Tween-20 + 10% BSA + 1% NP40. Anti-*D*E-cadherin staining was performed following [12]. In brief, ovaries were dissected in Schneider's insect medium (Biowest L0207-500), fixed in 1xPBS + 0.1% Tween-20 + 4% FA for 15 min, washed with PBS-T and incubated in 0.5% goat serum diluted with PBS-T for 1 hour.

Primary anti-*D*E-cadherin and its secondary antibodies were incubated at 4°C overnight, then washed with PBS-T. Primary antibodies were used at the following concentrations: mouse anti-Hts (1B1) (Developmental Studies Hybridoma Bank [DSHB], University of Iowa), 1:100; rabbit anti-Vasa (a gift from R. Lehmann), 1:3000; goat anti-GFP, FITC (Abcam, ab6662), 1:500; alpaca anti-GFP-Booster_Atto488 (ChromoTek, gba 488-100), 1:200; mouse anti–Lamin C (LC28.26-s) (DSHB), 1:30; rat anti-*D*E-cadherin (DCAD2) (DSHB), 1:50; guinea pig anti-Hh (raised against a His-tagged fragment of the Hh protein (aa. 82-257) produced in *E. coli*; polyclonal antibodies were affinity purified), 1:100; rabbit anti-phospho-Mad (Abcam, 52903), 1:1000; rabbit anti-cleaved Dcp-1 (Cell Signaling Technology, 9578), 1:100; guinea pig anti-Nuf [13], 1:500; mouse anti–Ptc (Apa-1) (DSHB), 1:100; mouse anti-Rab11 (BD Biosciences), 1:100. Secondary antibodies (FITC from Abcam and Cy2-, Cy3- and Cy5-conjugated from Jackson ImmunoResearch) were used at 1:100. DNA staining was performed using the DNA dye Hoechst (Sigma B2883 10mg/ml in H_2O) at 1:1000 in PBT.

Imaging and Image Analysis

Images were captured with Leica SPE or SP5 confocal microscopes controlled by the Leica LAS AF software and using the 63x oil immersion objective. In most experiments Z-sections were taken every 0.7 µm. Images were analysed utilising ImageJ and processed with Adobe Photoshop and Adobe Illustrator. To analyse GSC numbers, image series were acquired in the SPE confocal microscope at 400 or 600 Hz, zooming in the germarium. To analyse fluorescence intensity, z-series of control and experimental samples were acquired in the SP5 confocal microscope at 400 Hz with exactly the same settings. Average fluorescence intensity per pixel was quantified with the Image J "Region of Interest manager" analysis tool. In general, to quantify fluorescent intensity we created a MAX projection of the CpC cluster and drew approx. 15 small squares (of ~0.1-0.5 microns^2 each) per germarium that contained regions of bright signal. The average pixel intensity was calculated and plotted. Average background levels were measured in each germarium with at least 5 boxes in GSC nuclei or cytoplasm. To quantify Ptc levels, we obtained the MAX projection image from 5-6 z-sections and scored overall fluorescent signal of areas of similar size (approx. 1200 microns^2 each) in the anterior half of the germarium. To quantify *D*E-cadherin::GFP we measured the

fluorescent signal in both cap cell-cap cell and cap cell-GSC interfaces. To quantify pMad levels in GSCs, we obtained MAX projections for individual GSCs and scored fluorescence intensity in a minimum of 5 boxes of similar areas (~1-2 micron^2 each) per projection. Average background levels were measured in each germarium with at least 5 boxes in the nucleus or cytoplasm of older cysts (which do not express pMad).

Ex vivo ovariole culture was performed following [14]. In short, ovarioles were isolated in Schneider medium supplemented with 10% Foetal Bovine Serum (Sigma, F3018), 0.6% (V/V) streptomycin/penicillin antibiotic mix (Invitrogen, 15140-122) and 0.20 mg ml-1 insulin (Sigma, 15500). Individual ovarioles were transferred to a 35 mm Poly-d-lysine Coated plate (Mattek, P35GC-1.5-10-C) containing supplemented Schneider medium.

Fluorescence Recovery After Photobleaching (FRAP)

Z-sections were taken every 0.3 µm. Pre-bleach z-stacks were collected with the 488 nm laser line at 20% power. The pre-bleach signal was considered 100% fluorescent intensity. One or several regions of interest per niche which corresponded to CpC-CpC or CpC-GSC surfaces were bleached with the 488 nm and 405 nm laser lines at 100% power for three 2.6 second-long scans (400 Hz). The post-bleach signal was arbitrarily assigned a 0% value. Three Z-stacks were then collected at 10-min. time intervals. (400 Hz, 2 line average). Fluorescence intensity was quantified in single planes of the bleached regions in each time point.

Isolation of niche cells by Fluorescence-Activated Cell Sorting (FACS)

Preparation of single-cell suspensions were done optimizing a previous protocol [15]. In short, 120 ovary pairs were dissected in Schneider's insect medium (Biowest L0207-500) supplemented with 10% (v/v) heat-inactivated fetal bovine serum (Gibco 10500-064; S-FBS), washed twice with Cell Dissociation Solution Non-enzymatic 1x (Sigma C5914) and incubated with the digestion solution (670µl Trypsin 1X [SIGMA T4299] + 330µl Collagenase [SIGMA C9407] 10 mg/ml in PBS + 100µ DNAse I [Sigma AMPD1-1KT] + 100µl DNAse I buffer [Sigma AMPD1-1KT]) at 25°C for 15 min, rolling at 40 rpm in a dissecting well (NOTE: prior to treatment, ovaries were teased apart manually with the dissecting forceps). Ovaries were then mechanically disaggregated in a blue pipette tip and transferred to a FACS tube (BD

Falcon 352054). Trypsin and DNAse were inactivated with 1ml S-FBS and 100 µl Stop Solution, respectively. The supernatant was filtered through a 70µm nylon mesh and cells were collected by centrifugation at 420 x g for 5 minutes (4°C) and re-suspended in 1ml S-FBS. The resulting cell suspension was filtered again through a 70µm nylon mesh and propidium iodide was added at a final concentration of 1:500.

To isolate terminal filament and cap cells (but not escort cells), the suspended cells were separated according to cell size (forward and side scatter plots), negative propidium iodide label and GFP-signal intensity in a FACSAria cell sorter (BD) equipped with a 100 µm nozzle and at low pressure (20 psi). Cells were sorted directly into 0.5 ml lysis buffer and snap frozen in liquid nitrogen. Typically, we could sort ~1,000 TFCs and CpCs per experiment.

Sample preparation, Microarray hybridization and data analysis

RNA from three biological replicas per each of the three time-points (1-, 3- and 4-week old samples) was isolated using magnetic beads from ~400 cells. cDNA synthesis, library preparation and amplification (Pico Profiling) are described elsewhere [16]. After reverse-transcription, each cDNA sample was added to an amplification mix that was subdivided into five equivalent parts for PCR amplification (26 cycles). Amplified cDNA collections were purified with the PureLink PCR Purification Kit (ThermoFisher Scientific), resuspended in 40µl and their concentrations measured using a Nanodrop 1000 spectrophotometer. The nine cDNA collections were hybridized to GeneChip *Drosophila* Genome 2.0 Arrays from Affymetrix. Raw data (CEL files) generated with Affymetrix's AGCC software were used as input for the DTT software. For the resulting datasets, RMA (Robust Microarray Average) normalization and linear modelling by *limma* (Linear Models for Microarray Analysis) was performed. The p-value for the False Discovery Rate (FDR) was ≤ 0.05.

Quantification of mRNA levels by qPCR

RNA was isolated from ~20 ovaries with RNAeasy Micro (Qiagen 74004) and QIAshredder (Qiagen 79654) columns. To synthesize cDNA, 10 µl of mRNA were incubated for 5 min at 65°C with 1 µl (0.5 ⍰g) of Oligo(dT)$_{23}$ Anchored, (Sigma O4387) + 1 µl of 10mM dNTP mix and then on ice for 1 min. 4 µl of 5x First Strand buffer (Y02321) + 1 µl RNaseOUT Recombinant

Ribonuclease Inhibitor (40 units/ µl) (100000840) + 2 µl 0.1 M DTT (Y00147) from Invitrogen were added and incubated for 1 min at 42°C. Next, 1 µl of Super Script II RT from Invitrogen (18064-014) was added and incubated for 50 min at 42°C, 15 min at 70°C and then on ice for 1 min. Finally, mix was incubated for 20 min at 37°C with 1 µl of RNase H from Invitrogen (18021-014). Primers used were (5'-3'): *RpL32*, F: ATGACCATCCGCCCAGCATAC, R: GCTTAGCATATCGATCCGACTGG; β-*Tub*, F: GCAGTTCACCGCTATGTTCA , R: CGGACACCAGATCGTTCAT; *mam* long isoforms, F: ACAATCGACCACCACAACGC, R: TCGCCACTCAAATGCAGATGC; *mam* short isoform, F: AAATCTTCGCCCAAGCCAGC, R: TGCCGGTATTGGAGCCATTG. The relative mRNA amounts of *mam* long and short isoforms were determined by real-time quantitative PCR (CFX Connect, Bio-Rad) using the comparative cycle threshold (C_T) method [17] and the EVAGreen supermix kit (Bio-Rad 1864033). Ribosomal protein L32 (*RpL32*) and β-Tubulin (β-*Tub*) were used for normalisation. qPCR reactions contained 5 µl of SYBR *Premix Ex Taq* (Tli RNaseH Plus) kit from Takara (RR420A), 1 µl of template cDNA, 2 µl of F+R primers and 2 µl distilled water. The PCR conditions were: 95°C for 10 min (1x), 95°C for 15 sec and 67°C for 45 sec (40x). Ct values obtained resulted from three biological replicas and three technical replicas.

Quantification of mRNA levels by droplet digital PCR

Pico profiled samples from sorted niche cells (terminal filament cells and cap cells; see above) were used to quantify mRNA levels of *mam* long and short isoforms by droplet-digital PCR (ddPCR). Primers used were (5'-3'): *RpL32*, F: ATGACCATCCGCCCAGCATAC, R: GCTTAGCATATCGATCCGACTGG; β-*Tub*, F: GCAGTTCACCGCTATGTTCA , R: CGGACACCAGATCGTTCAT; *mam* long isoforms, F: ACAATCGACCACCACAACGC, R: TCGCCACTCAAATGCAGATGC; *mam* short isoform, F: CGGACCAAGTCTACCAACGC, R: TGTCAGGGCCATTGTCAACA. *RpL32* was used for normalisation. Each reaction contained 10 µl of Master Mix ddPCR EVAGreen (Bio-Rad), 150 nM of each primer and 2,5 ng of cDNA template (except in the case of the housekeeping control, where we used 0,5 ng). Samples were prepared in duplicate with 10% additional volumen. Droplet generation, PCR amplification and droplet analysis were done using the QX200 AutoDG ddPCR system (Bio-Rad). PCR conditions were: 95°C for 5 min (1x), 95°C for 1 min and 65.2°C for 2 min (44x). For

all steps a ramp rate of 2°C/s was used. Data were analysed with the Quantasoft software 1.7.4.0917 (Bio-Rad).

Statistical Analysis

Samples were collected from at least 5 different adult females grown in similar conditions. Graphs represent the mean values and standard error of each dataset. Fluorescence arbitrary units (AU) were normalised to controls. P-values were obtained using a two-tailed Student's t-test to determine values that were significantly different (*: $P \leq 0.05$, **: $P \leq 0.005$, ***: $P \leq 0.0005$). In all graphs, bar comparisons without asterisks indicate non-significant differences.

Supplemental References

1. Bolívar J, Pearson J, López-Onieva L, González-Reyes A. Genetic dissection of a stem cell niche: the case of the Drosophila ovary. Dev Dyn. 2006;235(11):2969-79. PubMed PMID: 17013875.

2. Tanimoto H, Itoh S, ten Dijke P, Tabata T. Hedgehog creates a gradient of Dpp activity in Drosophila wing imaginal discs. Mol Cell. 2000;5:59-71.

3. Bolivar J, Huynh JR, Lopez-Schier H, Gonzalez C, St Johnston D, Gonzalez-Reyes A. Centrosome migration into the Drosophila oocyte is independent of BicD and egl, and of the organisation of the microtubule cytoskeleton. Development. 2001;128(10):1889-97. PubMed PMID: 11311168.

4. Huang J, Zhou W, Dong W, Watson AM, Hong Y. Directed, efficient, and versatile modifications of the Drosophila genome by genomic engineering. Proc Natl Acad Sci U S A. 2009;106(20):8284-9. Epub 2009/05/12. doi: 0900641106 [pii]. 10.1073/pnas.0900641106. PubMed PMID: 19429710.

5. Martin AC, Gelbart M, Fernandez-Gonzalez R, Kaschube M, Wieschaus EF. Integration of contractile forces during tissue invagination. J Cell Biol. 2010;188(5):735-49. doi: 10.1083/jcb.200910099. PubMed PMID: 20194639; PubMed Central PMCID: PMCPMC2835944.

6. Torroja C, Gorfinkiel N, Guerrero I. Patched controls the Hedgehog gradient by endocytosis in a dynamin-dependent manner, but this internalization does not play a major role in signal transduction. Development. 2004;131(10):2395-408. Epub 2004/04/23. doi: 10.1242/dev.01102 [pii]. PubMed PMID: 15102702.

7. Helms W, Lee H, Ammerman M, Parks A, Muskavitch M, Yedvobnick B. Engineered Truncations in the Drosophila Mastermind Protein Disrupt Notch Pathway Function. Dev Biol. 1999;215:358-74.

8. Pan L, Chen S, Weng C, Call G, Zhu D, Tang H, et al. Stem cell aging is controlled both intrinsically and extrinsically in the Drosophila ovary. Cell Stem Cell. 2007;1(4):458-69. PubMed PMID: 18371381.

9. Sanson B, White P, Vincent J. Uncoupling cadherin-based adhesion from wingless signalling in Drosophila. Nature. 1996;383(17 October):627-30.

10. Li MA, Alls JD, Avancini RM, Koo K, Godt D. The large Maf factor Traffic Jam controls gonad morphogenesis in Drosophila. Nat Cell Biol. 2003;5(11):994-1000. doi: 10.1038/ncb1058. PubMed PMID: 14578908.

11. Rojas-Rios P, Guerrero I, Gonzalez-Reyes A. Cytoneme-mediated delivery of hedgehog regulates the expression of bone morphogenetic proteins to maintain germline stem cells in Drosophila. PLoS Biol. 2012;10(4):e1001298. Epub 2012/04/18. doi: 10.1371/journal.pbio.1001298. PBIOLOGY-D-11-02864 [pii]. PubMed PMID: 22509132.

12. Song X, Zhu CH, Doan C, Xie T. Germline stem cells anchored by adherens junctions in the Drosophila ovary niches. Science. 2002;296(5574):1855-7. Epub 2002/06/08. doi: 10.1126/science.1069871. 296/5574/1855 [pii]. PubMed PMID: 12052957.

13. Calero-Cuenca FJ, Espinosa-Vázquez JM, Reina-Campos M, Díaz-Meco MT, Moscat J, Sotillos S. Nuclear fallout provides a new link between aPKC and polarized cell trafficking. BMC Biology. 2016;14(1). doi: 10.1186/s12915-016-0253-6.

14. Díaz de la Loza MC, Díaz-Torres A, Zurita F, Rosales-Nieves AE, Moeendarbary E, Franze K, et al. Laminin Levels Regulate Tissue Migration and Anterior-Posterior Polarity during Egg Morphogenesis in Drosophila. Cell Reports. 2017;20(1):211-23. doi: 10.1016/j.celrep.2017.06.031.

15. Lim RS, Osato M, Kai T. Isolation of undifferentiated female germline cells from adult Drosophila ovaries. Curr Protoc Stem Cell Biol. 2012;Chapter 2:Unit2E 3. doi: 10.1002/9780470151808.sc02e03s22. PubMed PMID: 22872426.

16. Gonzalez-Roca E, Garcia-Albéniz X, Rodriguez-Mulero S, Gomis RR, Kornacker K, Auer H. Accurate Expression Profiling of Very Small Cell Populations. PLoS ONE. 2010;5(12):e14418. doi: 10.1371/journal.pone.0014418.

17. Livak KJ, Schmittgen TD. Analysis of relative gene expression data using real-time quantitative PCR and the 2(-Delta Delta C(T)) Method. Methods. 2001;25(4):402-8. Epub 2002/02/16. doi: 10.1006/meth.2001.1262. S1046-2023(01)91262-9 [pii]. PubMed PMID: 11846609.

SUPPLEMENTARY TABLE 1 (related to Figure 1 and Figure S1)

Linear Fold Change	Increase/ Decrease	*p* value FDR	Gene Symbol
**3 weeks *vs* 1 week**			
8.615	Decrease	0.045	*rdo*
5.947	Decrease	0.027	CG34259
5.067	Decrease	0.033	CG13405
4.717	Decrease	0.007	CG10013
3.796	Decrease	0.033	*hiw*
3.105	Decrease	0.013	*ru*
2.718	Decrease	0.009	*FucTD*
2.673	Decrease	0.033	CG5080
3.588	Increase	0.033	*mam*
**4 weeks *vs* 1 week**			
7.835	Decrease	0.022	*rdo*
7.157	Decrease	0.035	CG5707
6.787	Decrease	0.024	CG12744
6.539	Decrease	0.048	*Phf7*
5.670	Decrease	0.035	*yin*
5.558	Decrease	0.022	CG10195
5.347	Decrease	0.039	*Fili*
4.638	Decrease	0.047	*RpS5b*
4.541	Decrease	0.025	CG14434
4.140	Decrease	0.022	CG13405
3.995	Decrease	0.012	CG10013
3.874	Decrease	0.026	CG34259
3.615	Decrease	0.031	CG30001
3.506	Decrease	0.022	*Acer*
3.375	Decrease	0.022	CG34200
3.297	Decrease	0.012	CG5080
2.884	Decrease	0.035	CG11858
2.851	Decrease	0.012	CG32803
2.679	Decrease	0.020	*Cap-G*
2.589	Decrease	0.015	*ru*
2.567	Decrease	0.035	*Smu1*
2.470	Decrease	0.029	CG9203
2.377	Decrease	0.035	CG6629
2.360	Decrease	0.038	*RhoGEF4*
2.326	Decrease	0.047	*ko*
2.279	Decrease	0.012	*FucTD*
2.202	Decrease	0.040	CG10903
2.120	Decrease	0.035	*l(2)k14505*
2.088	Decrease	0.022	*nxf4*

2.051	Decrease	0.035	*TppII*
2.049	Decrease	0.047	CG1136
2.034	Decrease	0.035	*mRpL41*
5.227	Increase	0.020	CG11893
4.541	Increase	0.048	CG8620
3.700	Increase	0.019	CG43111
3.545	Increase	0.012	*Hsp22*
3.474	Increase	0.012	*GV1*
3.323	Increase	0.020	*l(2)41Ab*
3.254	Increase	0.048	*TpnC25D*
3.206	Increase	0.012	*cenG1A*
3.070	Increase	0.018	*dm*
2.996	Increase	0.016	CG30421
2.958	Increase	0.020	CG30031 /// CG4269
2.957	Increase	0.026	*Pvf2*
2.903	Increase	0.014	CG5953 / *gag-int-pol*
2.873	Increase	0.026	*Arc2*
2.765	Increase	0.020	CG18547
2.718	Increase	0.024	CG34301
2.677	Increase	0.035	*Rcd2*
2.520	Increase	0.035	*clumsy*
2.473	Increase	0.047	*mam*
2.471	Increase	0.047	*Gadd45*
2.467	Increase	0.022	CG31710
2.442	Increase	0.020	CG40005 /// *cta*
2.435	Increase	0.022	*Spn42Da*
2.434	Increase	0.031	*Cpr92A*
2.406	Increase	0.012	*MESK2*
2.388	Increase	0.039	*MRP*
2.386	Increase	0.023	CG15784
2.379	Increase	0.022	CG11447
2.044	Increase	0.033	*Hsp70Bb /// Hsp70Bc*
2.033	Increase	0.035	*Hsp70Ba /// Hsp70Bb /// Hsp70Bbb /// Hsp70Bc*

Table S1. List of genes whose expression is either up- or down-regulated during ageing in terminal filament and cap cells. Expression levels of 1-week old cells have been compared to cells isolated from 3- or 4-week old ovaries. *p* value for the FDR (False Discovery Rate) was set <0.05. Linear Fold Change was set >2.

SUPPLEMENTARY FIGURE LEGENDS

Supplementary Figure S1 (related to Figure 1): Graphical representation of genes up- or down-regulated during ageing in niche support cells. Numerical values of identified differences in expression and the *p* values of False Discovery Rates can be found in Table S1.

Supplementary Figure S2 (related to Figure 2): Effect of *mam* mRNA reduction on GSC numbers and Hedgehog and DE-cadherin levels. Overexpression of *mam* short isoform in somatic cells of the ovary does not affect GSC numbers. Overexpression of *mam* long isoform in somatic cells of the ovary does not affect *mam* short isoform levels. Additional controls to discard the effect of different chromosomal backgrounds on GSC numbers. (A) A slight reduction in *mam* mRNA levels does not affect significantly GSC numbers whereas a stronger reduction decreases them. **(B)** Quantification of Hh and *D*E-cad levels in *bab1>mam RNAi* grown at 22ºC and kept for 4 weeks at 22ºC. **(C)** Graph showing that females carrying either the TM6B balancer chromosome or the recombinant chromosome with the *bab1-Gal4, tub-Gal80^{ts}* constructs contain similar GSC numbers. **(D)** Graph showing that *bab1>mam^{Short}* does not induce GSC loss. **(E)** Real time quantitative PCR data showing that overexpression of *mam* long isoform in somatic cells of the ovary (using the *traffic-jam* Gal4 driver) induces high levels of *mam^{Long}* specifically, without affecting *mam^{Short}* isoform expression. We used two different housekeeping genes to normalise our measurements, β-tubulin and RpL32. **(F)** Graph showing that females carrying the *tub-Gal80^{ts}* construct and the TM6B balancer chromosome do not show differences in GSC numbers with females carrying the *tub-Gal80^{ts}* construct and the *hh-* or *ptc-Gal4* lines. Numbers in bars represent germaria analysed (n). Error bars are shown for the different samples.

Supplementary Figure S3 (related to Figures 2 and 3): *mam* overexpression in niche cells does not induce GSC apoptosis. (A) Z-projection of germaria from young (1 week) and aged (4 weeks) flies stained with antibodies to Hts (red; to distinguish germline cells and cysts) and Dcp1 caspase (green; to label apoptotic cells). Dcp1 expression is indistinguishable in young (n=22) *vs.* aged germaria (n=20). **(B)** Z-projection of germaria from control and experimental flies overexpressing *mam* for 1 week stained with antibodies to Dcp1 caspase (to label

apoptotic cells) and with the DNA dye Hoechst (blue). Dcp1 expression is indistinguishable in control (n=6) *vs.* experimental germaria (n=10). Dashed lines outline Dcp1 positive cells or cell clusters, which never corresponded to GSCs. Scale bars 5 µm.

Supplementary Figure S4 (related to Figure 3): *mam* overexpression reduces *D*E-cadherin levels. (A) Z-projection of germaria from *bab1>mam* flies kept for two weeks at $29^{o}C$ stained to visualise DNA and the endogenous *D*E-cadherin protein. **(B)** Quantification of *D*E-cadherin levels shows they decrease in experimental niches. At least 15 measurements per germarium were taken. **(C)** *D*E-*cadherin* overexpression for two weeks in niche cells does not increase GSC numbers and it cannot rescue the GSC loss produced by *mam* overexpression. Numbers in bars represent germaria analysed. Dashed lines outline CpC clusters. Scale bar 5 µm.

Supplementary Figure S5 (related to Figure 4): Effect of *mam* overexpression on *D*E-cadherin::GFP recycling and of ageing and *mam* over-expression on Nuf levels in CpCs. **(A)** Images showing *D*E-cadherin::GFP FRAP in 1-week old CpCs from control and *bab1>mam* niches. **(B)** Both conditions show similarly low fluorescence recoveries. **(C)** Z-projection of germaria from 1-week and 6-week old flies stained to visualise TFC and CpC nuclear membranes (Lamin-C; green) and the Nuf protein (red). **(D)** Nuf levels decrease with age in niche cells. **(E)** Z-projection of germaria from *bab1>mam* flies kept for two weeks at $29^{o}C$ stained to visualise TFC and CpC nuclear membranes (Lamin-C; green) and the Nuf protein (red). **(F)** Nuf levels do not change in experimental CpCs. Numbers in bars represent germaria analysed. Data plotted in (D, F) were obtained from at least 15 measurements/germarium. Purple dashed boxes in (A) demarcate the photobleached regions; dashed lines in (C, E) outline CpC clusters. n= number of germaria analysed. Pre-bleach levels are considered 100% intensity and the post-bleach signal is arbitrarily considered 0%. Later time points are referenced to the post-bleach values.

Supplementary Figure S6 (related to Figure 5): *dpp* signalling in aged and *mam*-overexpressing niches. (A) Z-projections of germaria from 1-week and 6-week old flies stained to visualise pMad (red) and spectrosomes (Hts; green). **(B)** Quantification of pMad

levels in (A). **(C, D)** Similar experiments to (A, B) but comparing control and experimental flies overexpressing *mam* for 2 weeks. **(E)** Graph depicting the quantification of GSC numbers in control and experimental conditions in which *mam* and/or *hh::GFP* are overexpressed. *hh* overexpression does not induce extra GSCs. 2-week *hh* overexpression in niche cells cannot rescue the GSC loss produced by high *mam* levels. Dashed lines outline GSCs. Numbers in bars represent germaria analysed. Scale bars 5 µm.

Supplementary Figure S7 (related to Figure 6): *SOD1* overexpression does not affect GSC numbers. (A) Z-projections of germaria from control and experimental flies overexpressing *SOD1+mam* for 2 weeks stained to visualise Hh (red), *D*E-cad (green) and DNA (blue). **(B)** Quantification of Hh and *D*E-cad levels in (A). **(C)** Quantification of GSC numbers in control and *SOD1*-overexpressing niches. Numbers in bars represent germaria analysed. Dashed lines outline CpC clusters. Scale bars 5 µm.

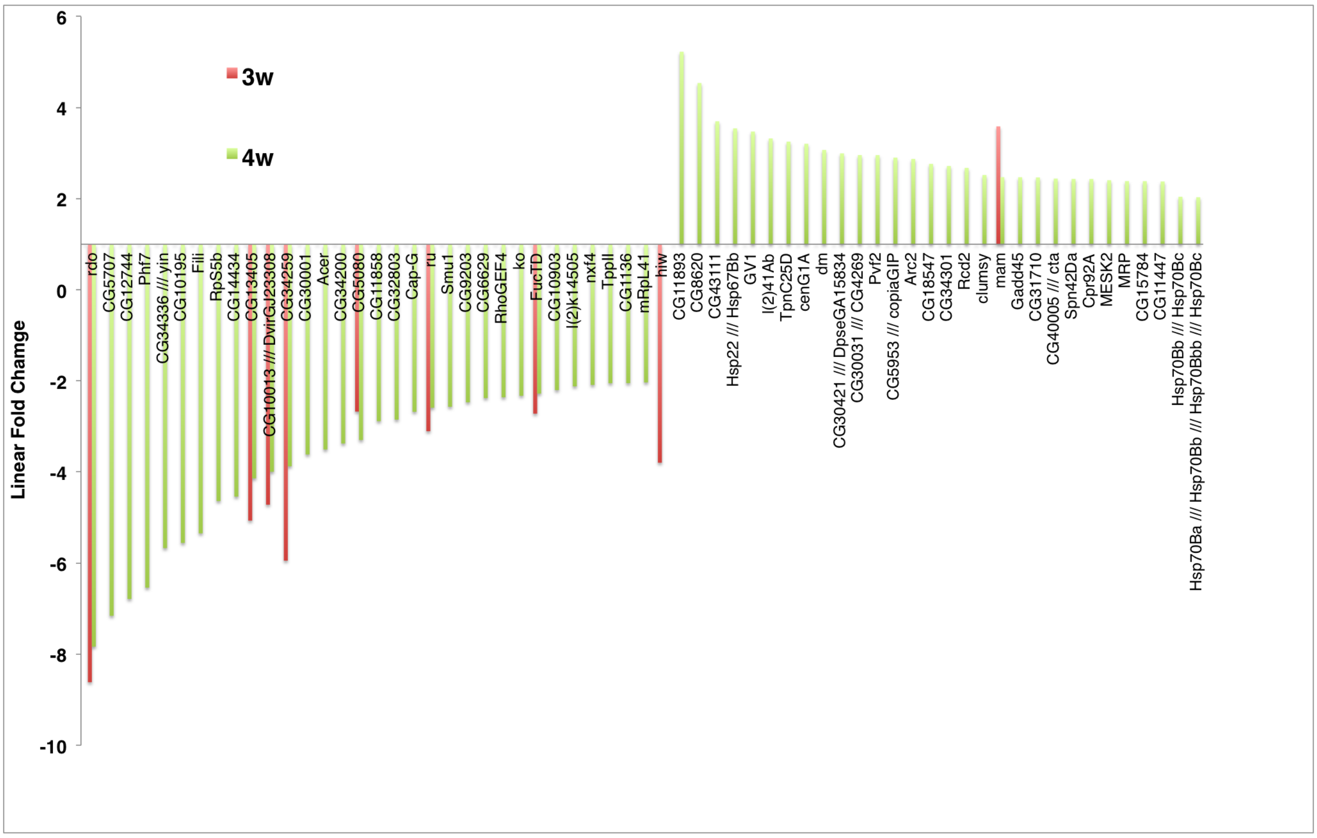

Fig. S1

Lobo-Pecellín et al.

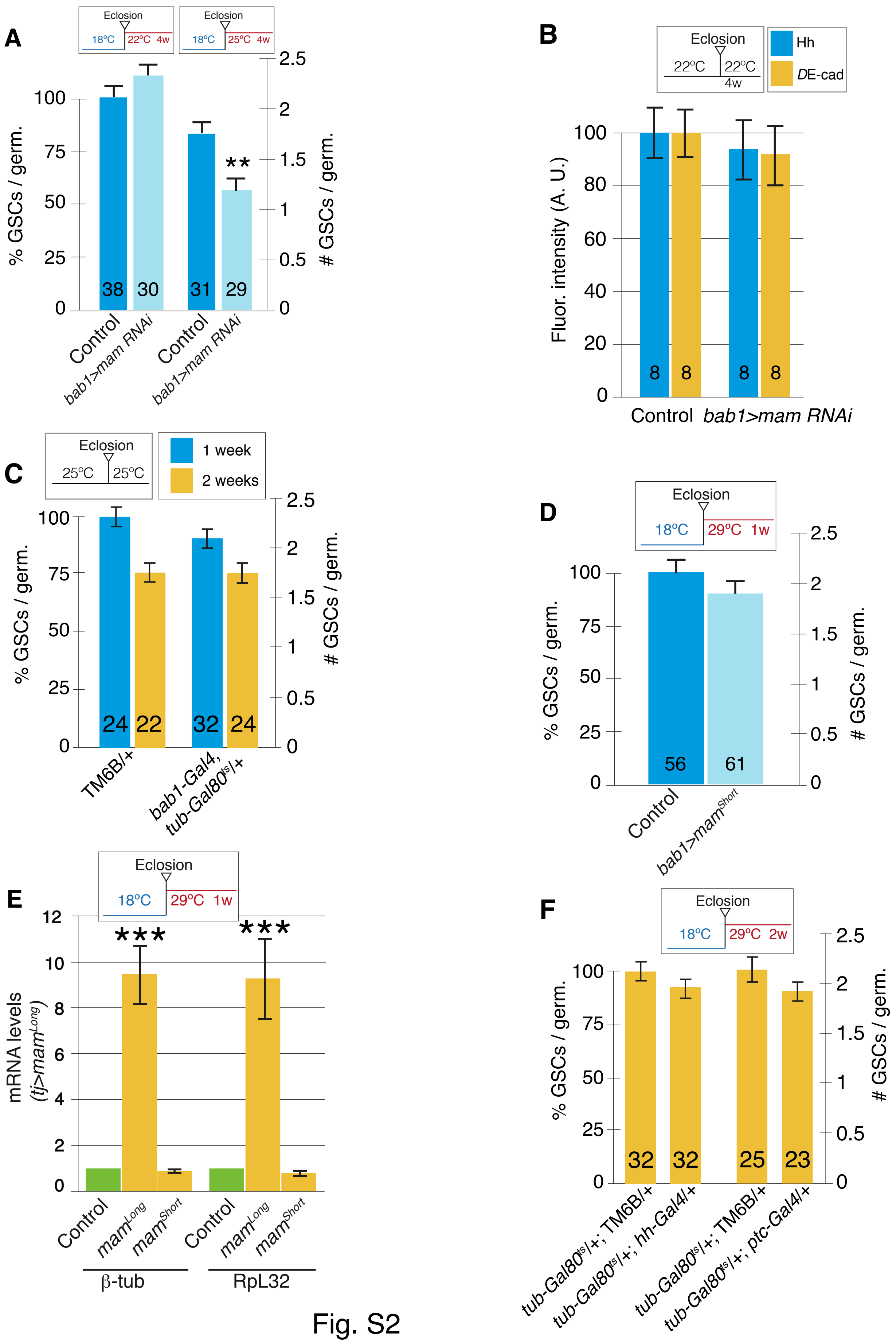

Fig. S2

Lobo-Pecellín et al.

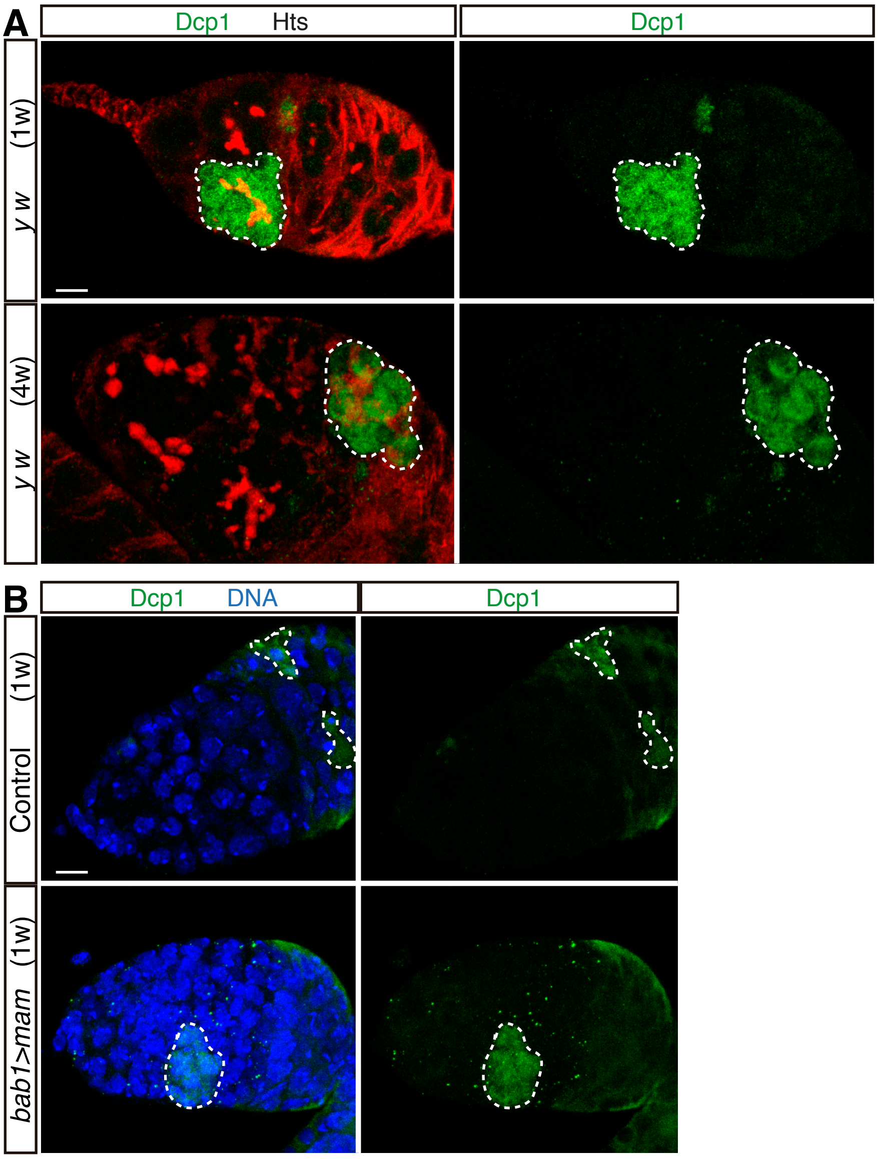

Fig. S3

Lobo-Pecellín et al.

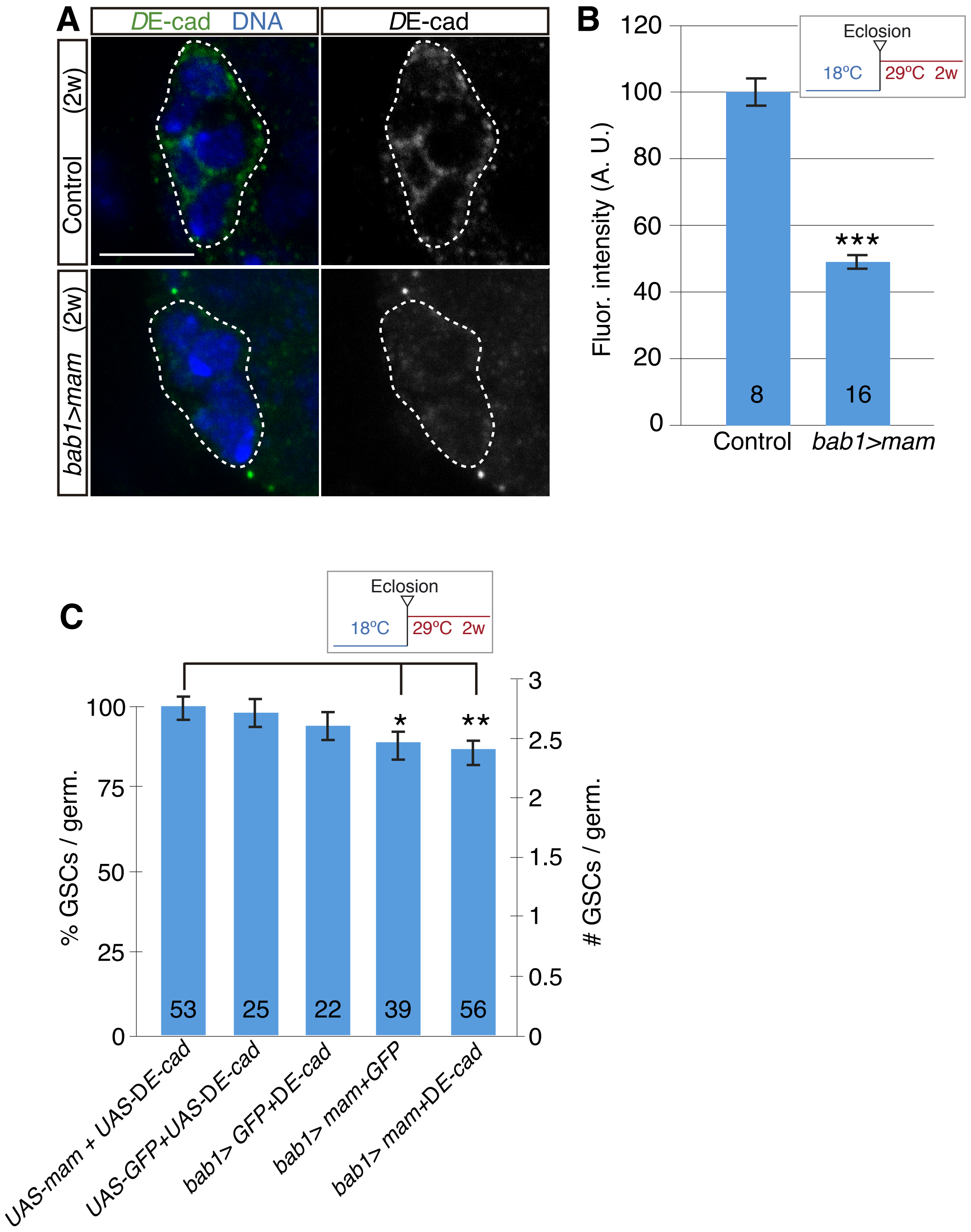

Fig. S4

Lobo-Pecellín et al.

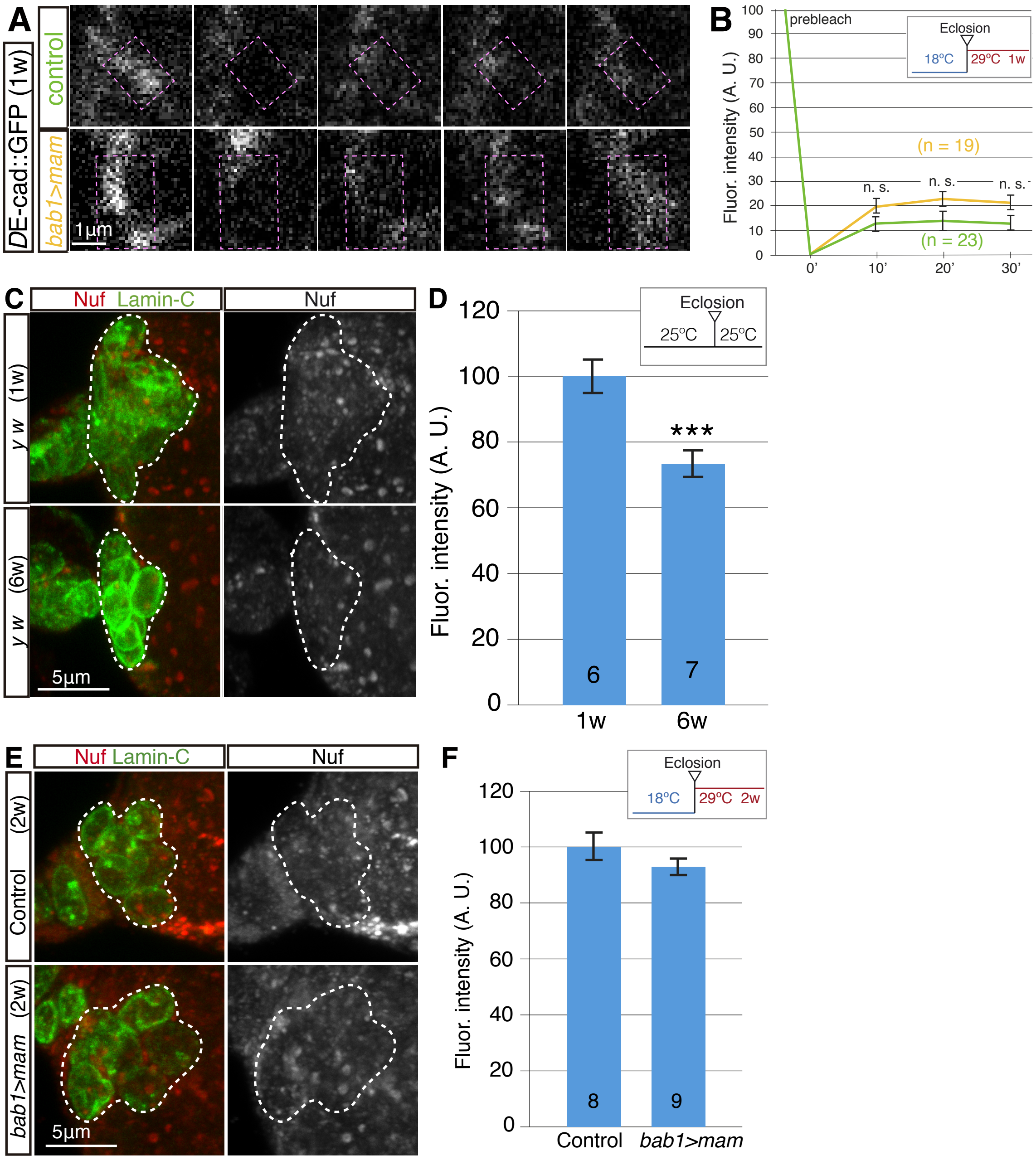

Fig. S5

Lobo-Pecellín et al.

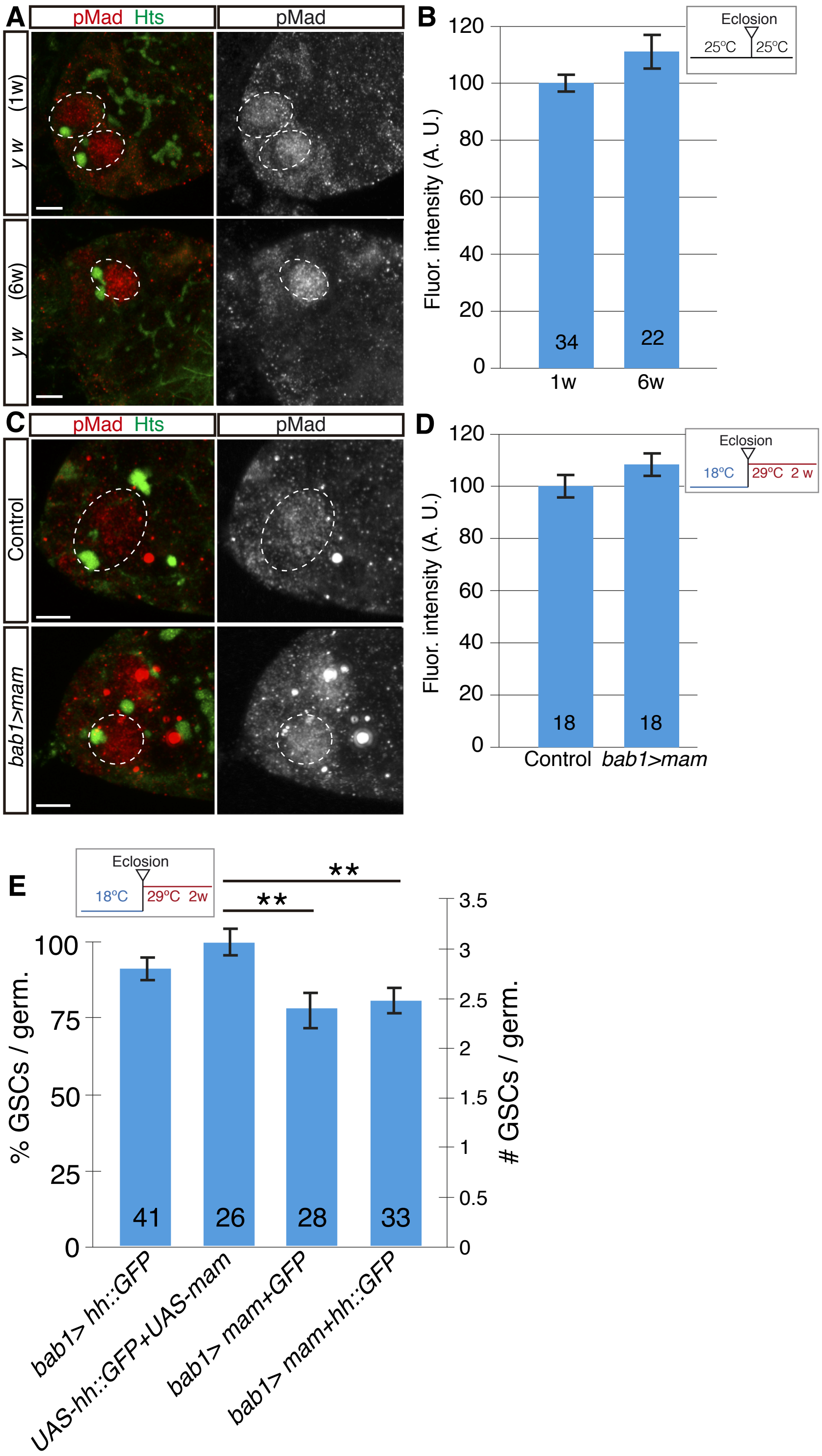

Fig. S6
Lobo-Pecellín et al.

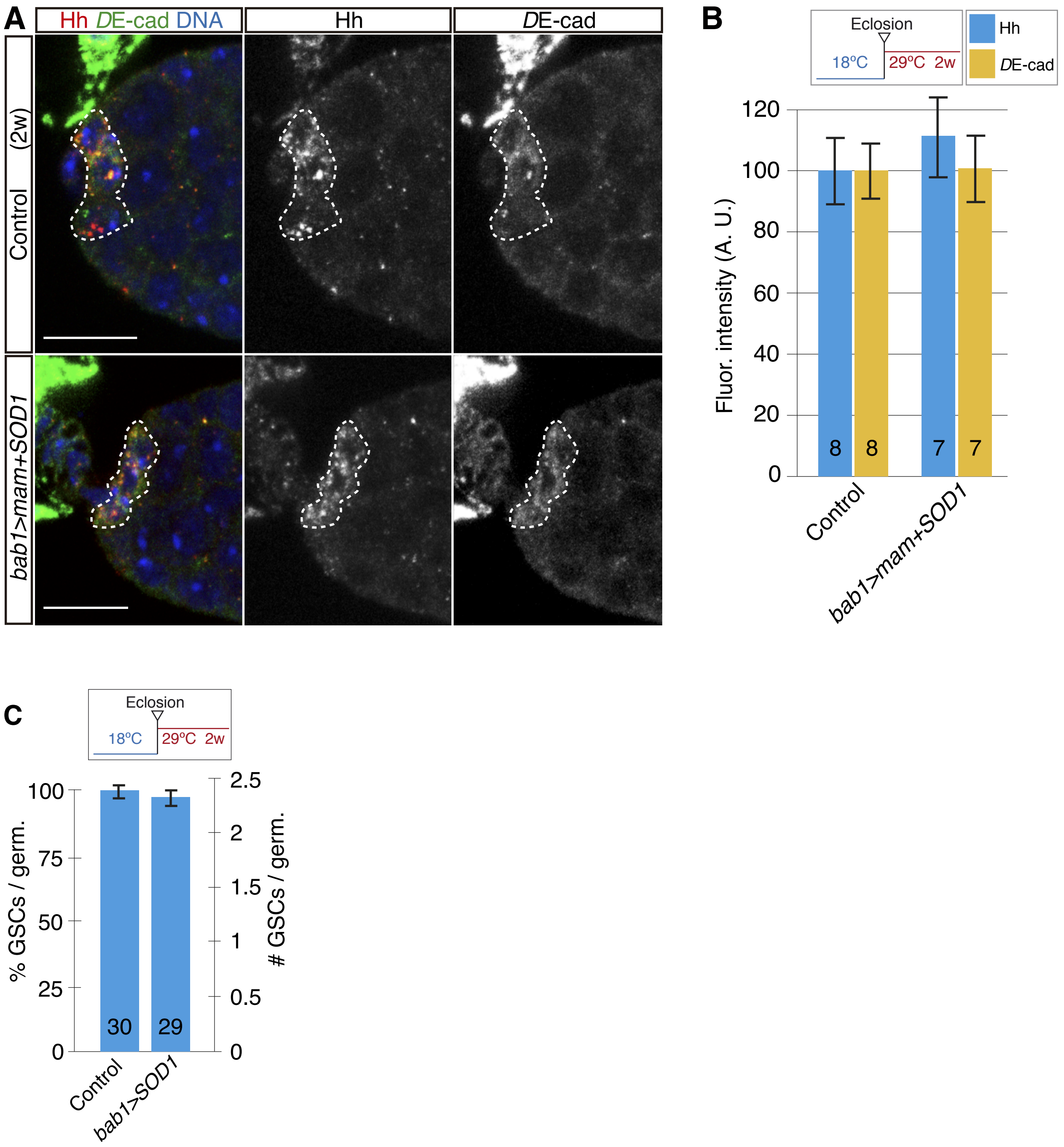

Fig. S7
Lobo-Pecellín et al.
